# Changes in the Mitochondria-Related Nuclear Gene Expression Profile during Human Oocyte Maturation by the IVM Technique

**DOI:** 10.3390/cells11020297

**Published:** 2022-01-16

**Authors:** Zhi-Yong Yang, Min Ye, Ya-Xin Xing, Qi-Gui Xie, Jian-Hong Zhou, Xin-Rui Qi, Kehkooi Kee, Ri-Cheng Chian

**Affiliations:** 1 Center for Reproductive Medicine, Shanghai Tenth People’s Hospital, Tongji University School of Medicine, Shanghai 200072, China; yangzysh@163.com (Z.-Y.Y.); xyx15800931730@163.com (Y.-X.X.); 2 Department of Human Anatomy, Histology and Embryology, Tongji University School of Medicine, Shanghai 200092, China; xinruiqi@tongji.edu.cn; 3 Center for Stem Cell Biology and Regenerative Medicine, Department of Basic Medical Sciences, School of Medicine, Tsinghua University, Beijing 100084, China; yemin@mail.tsinghua.edu.cn; 4 Department of Obstetrics and Gynecology, Shanghai Tenth People’s Hospital, Tongji University School of Medicine, Shanghai 200072, China; cykstar@163.com (Q.-G.X.); 13918173861@163.com (J.-H.Z.)

**Keywords:** human oocyte, in vitro maturation, IVM, mitochondria, gene expression, RNAseq, oocyte maturation, cytoplasmic maturation

## Abstract

To address which mitochondria-related nuclear differentially expressed genes (DEGs) and related pathways are altered during human oocyte maturation, single-cell analysis was performed in three oocyte states: in vivo matured (M-IVO), in vitro matured (M-IVT), and failed to mature in vitro (IM-IVT). There were 691 DEGs and 16 mitochondria-related DEGs in the comparison of M-IVT vs. IM-IVT oocytes, and 2281 DEGs and 160 mitochondria-related DEGs in the comparison of M-IVT vs. M-IVO oocytes, respectively. The GO and KEGG analyses showed that most of them were involved in pathways such as oxidative phosphorylation, pyruvate metabolism, peroxisome, and amino acid metabolism, i.e., valine, leucine, isoleucine, glycine, serine, and threonine metabolism or degradation. During the progress of oocyte maturation, the metabolic pathway, which derives the main source of ATP, shifted from glucose metabolism to pyruvate and fatty acid oxidation in order to maintain a low level of damaging reactive oxygen species (ROS) production. Although the immature oocytes could be cultured to a mature stage by an in vitro technique (IVM), there were still some differences in mitochondria-related regulations, which showed that the mitochondria were regulated by nuclear genes to compensate for their developmental needs. Meanwhile, the results indicated that the current IVM culture medium should be optimized to compensate for the special need for further development according to this disclosure, as it was a latent strategy to improve the effectiveness of the IVM procedure.

## 1. Introduction

It has been 40 years since the first in vitro fertilization–embryo transfer (IVF-ET) baby was born into the world [1]. Up to now, this landmark technology has greatly improved infertility medical care and has made huge strides and fast progress toward finding suitable treatment options for infertile couples. The key issue of this technique is to obtain mature oocytes and fair-quality embryos; however, about 15–29% of oocytes are immature and are discarded in IVF-ET treatment [2,3]. The in vitro mature (IVM) technique is a very important complementary technique to utilize immature oocytes harvested in assisted reproductive treatment (ART). The initial basic work of IVM started in 1935, using rabbit ova, by Pincus and Enzmann [4], and in the 1960s Edwards made significant inroads into IVM in humans [5]. It was not until 1991 that the first baby was born using immature oocytes collected from an unstimulated cycle in a donor oocyte program [6], and in 1994 occurred the first birth from an untreated polycystic ovarian patient using her own oocytes [7]. In the next two decades, IVM techniques were systematically developed. In 2021, the practice committees of the American Society for Reproductive Medicine (ASRM), the Society of Reproductive Biologists and Technologists, and the Society for Assisted Reproductive Technology (SART) presented a considerable body of published evidence supporting the conclusion that IVM should no longer be considered an experimental technique [8]. However, to date, in spite of thousands of babies being born as a result of IVM technology, the pregnancy rate in terms of efficiency of IVM treatment is still lower than the mainstream of treatment, in which oocytes are derived from in vivo maturation following conventional ovarian stimulation [9,10]. It was believed that IVM oocytes are suboptimal due to the IVM procedure. For two decades, many efforts have been made to improve the IVM technique, including different culture systems and protocols [11]. It is important to identify whether or not there will be the differences between in vivo and in vitro matured oocytes in order to find the right approach for clinical practice. In 2016, one morphological ultrastructure study using transmission electron microscopy compared matured oocytes in vitro with those matured in vivo, and found that both in vivo and in vitro matured oocytes were largely, but not entirely, similar [12]. In 2020, we used a single-cell multiomic analysis method to demonstrate that both the global transcriptomic profiles and the aneuploidy ratio were similar, and furthermore some differentially expressed genes (DEGs) were found when comparing these two kinds of matured oocytes. The DEGs were mainly related to mitochondrial respiration biogenesis and energy production [13]. Meanwhile, another study for further assessment of the subcellular distribution of mitochondria in cytoplasm during in vitro meiotic maturation disclosed the pattern of dynamic changes in mitochondrial distribution after germinal vesicle breakdown (GVBD), which is an important event in nuclear maturation [14]. Although the authors tried to establish a linkage between nuclear maturation and cytoplasmic maturation as mitochondria-generating ATP plays key roles in maturation process, it was difficult to illuminate these morphological changes relevant to the function from the perspective of structure alone. Thus, the present work aims at exploring the expression profile changes of mitochondria-related genes during human oocyte maturation using the IVM technique.

## 2. Materials and Methods

### 2.1. Ethical Approval

This study was approved by the Ethics Committee of the Tongji University School of Medicine (approval code: 2017YXY001). All procedures performed in studies involving human participants were in accordance with the ethical standards of the institutional and/or national research committee and with the 1964 Helsinki Declaration and its later amendments or comparable ethical standards. All individuals participating in this study signed informed consent for oocyte donation, in the case of patients who underwent IVF treatment who did not want to preserve surplus oocytes after delivering one or more healthy babies, and patients who underwent fertility preservation during Caesarean delivery operation who terminated the fertility preservation service and voluntarily contributed oocytes that were no longer needed.

### 2.2. Source of Oocytes

**Figure 1:**
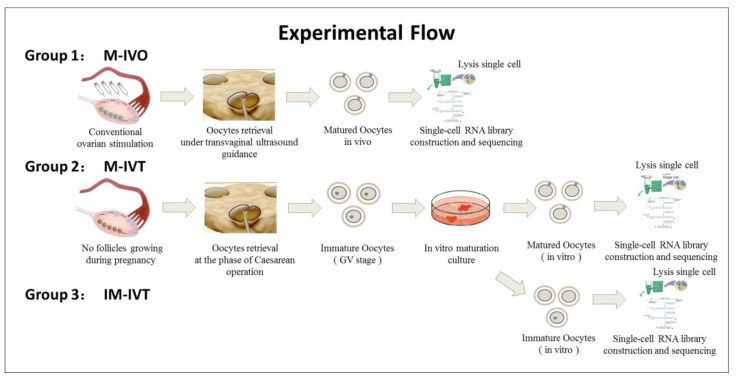
Schematic representation of the experimental procedures and group treatments.

The oocytes used in this study were from donations and were divided into three groups: (1) Matured oocytes in vivo (M-IVO, n = 8) and these oocytes were obtained from controlled ovarian stimulation undergoing assisted reproductive treatment in which seven patients received pituitary gland down regulation by GnRH agonist (Decapeptyl, Ferring GmbH, Kiel, Germany) and were followed by exogenous FSH (Gonal-F, Merck Serono, Darmstadt, Germany or Puregon, MSD, Kenilworth, NJ, USA) to stimulate more than one follicles to grow until at least three follicles with a diameter of ≥18 mm observed with ultrasound scan monitoring; oocyte retrieval was performed at 36–38 h after 10,000 IU hCG (Chorionic Gonadotrophin for Injection, Livzon Pharmacy, Zhuhai, Guangdong, China) administration. (2) Matured oocytes in vitro (M-IVT, n = 12), these oocytes were collected from six pregnant women undergoing Caesarean delivery, and then the immature oocytes were cultured to a mature stage in vitro (MII stage). (3) Immature oocytes (IM-IVT, n = 6), these oocytes were the remaining oocytes from four patients in group 2 that could not develop to the MII stage after 48 h of IVM. Figure 1 represents the schematic diagram of the experimental procedures and the oocyte sources.

### 2.3. Oocyte Retrieval Procedure

Immature oocytes were collected in the final phase of Caesarean operations. A 5 mL volume syringe with a 20 G needle was used to pierce the small follicles presented on the surface of an ovary and follicular fluid aspirates was checked to pick up the cumulus oocyte complexes (COCs) under a stereo microscope. The COCs were washed twice in Gamete Buffer medium (K-SIGB, COOK, Bloomington, IN, USA) at 37 °C and then transferred to in vitro maturation medium.

The mature oocytes in the IVO group were aspirated from follicles under transvaginal ultrasound guidance using a single lumen aspiration needle (K-OPAA-1835, 18-gauge, COOK) connected to a vacuum pump (pressure setting: 80–100 mmHg). The COCs were collected and washed in Gamete Buffer medium (K-SIGB, COOK) at 37 °C and transferred into fertilization medium (K-SIFM, COOK) overlaid with paraffin oil (ART-4008, Origio, Trumbull, CT, USA) at 6% CO_2_ and 5% O_2_ at 37 °C. Denudation was performed 2–4 h after oocyte collection. COCs were briefly exposed to 80 IU/mL hyaluronidase solution (90101, IrvineScientific, Santa Ana, CA, USA) for a maximum of 60 s, followed by mechanical removal of cumulus cells surrounding the oocytes. Denuded oocytes were checked and mature MII oocytes with a presence of the first polar body in the perivitelline space were cryopreserved according to the instruction of the Vitrification Kit (12284001F, Origio) for further analysis.

### 2.4. In Vitro Maturation

The COCs enclosed a germinal vesicle (GV-stage) oocyte surrounded by multiple and uninterrupted layers of cubical and tightly compacted cumulus cell due to lack of the internal gonadotropin-related stimulation. Such immature COCs were transferred into a 60 × 15 mm one-well dish (353653, Falcon, San Mateo, CA, USA) containing 1 mL IVM medium (ART-1600, Origio) supplemented with final concentration of 75 mIU/mL hMG (Menotropins, Livzon Pharmacy, Zhuhai, Guangdong, China) and 10% *v/v* synthetic serum (Serum Substitute Supplement, IrvineScientific, USA) at 37 °C in an incubator with an atmosphere of 5% CO_2_ and 5% O_2_ with high humidity. Twenty-four hours after maturation in in vitro culture, COCs were stripped for identification of oocyte maturity through exposure to 80 IU/mL hyaluronidase solution (90101, IrvineScientific). If the oocytes were matured at the MII stage, then they were cryopreserved and the remaining immature oocytes continued to be cultured for another 24 h. Forty-eight hours after oocyte retrieval, the remaining stripped oocytes were re-examined and, if any had matured, they were cryopreserved for further analyses and the residual immature oocytes were also cryopreserved for the IM-IVT group.

### 2.5. Single-Cell RNA Library Construction and Sequencing

The single-cell RNA library was constructed according to our previously published method [13]. Briefly, the zona pellucida of oocytes was removed using Tyrode’s solution and digested in lysis buffer (1× PCR buffer II (without MgCl_2_), 1.35 mM MgCl_2_, 0.45% NP40, 4.5 mM DTT, 0.18 U/μL SUPERase-In, 0.36 U/μL RNase inhibitor, Thermo Fisher Scientific, Waltham, MA, USA). The supernatant was vortexed sufficiently and then centrifuged at 1000× *g* for 5 min at 4 °C. The DNA was isolated by Dynabeads Myone Carboxylic Acid (Thermo Fisher Scientific) for methylation-seq. Then, the UP1 primer (ATATGGATCCGGCGCGCCGTCGACTTTTTTTTTTTTTTTTTTTTTTTT) was added to the RNA lysate. The lysate was incubated at 70 °C for 90 s and immediately placed on ice. Then, the cDNA was synthesized by SuperScript III reverse transcriptase (Thermo Fisher Scientific). The extra primer was removed by ExoSAP-IT (Thermo Fisher Scientific). The polyA tail was added by the TdT. The UP2 primer (ATATCTCGAGGGCGCGCCGGATCCTTTTTTTTTTTTTTTTTTTTTTTT) was ligated to the cDNA by PCR. The cDNA was amplified with 24 cycles of PCR. The sequencing library was constructed by NEBNext Ultra DNA Library Prep Kit (NEB, Ipswich, MA, USA) according to the instruction. The library was sequenced using the Illumina platform. All chemicals were purchased from Sigma Aldrich, St. Louis, Mo, USA, unless mentioned otherwise.

### 2.6. Single-Cell Methylation Library Construction and Sequencing

The single-cell methylation was constructed by the post-bisulfite adaptor tagging (PBAT) method [15,16,17,18]. Briefly, the isolated DNA was incubated in protease mix (1× Tris-EDTA, 20 mM KCl, 0.3% Triton-X 100, 1 mg/mL protease, 10 ng carrier RNA, 1/100 lambda DNA) at 50 °C for 3 h, 75 °C for 30 min. The bisulfite conversion was conducted as the instruction of the EZ DNA Methylation Kit (ZYMO Research, Irvine, CA, USA). The 6N-oligo1 (biotin-CTACACGACGCTCTTCCGATCTNNNNNN) was ligated to DNA fragments by Klenow exo–(NEB, Ipswich, MA, USA). The extra primer was removed by exo-I (NEB, USA), then the DNA was purified using 0.8× AMPure XP beads (Beckman Coulter Life Sciences, USA). The biotin-labeled DNA fragment was isolated by Dynabeads M280 Streptavidin (Thermo Fisher Scientific, USA), then the second strand was synthesized by Klenow exo–. The library was amplified by 13 cycles of PCR. The library was purified by 0.8× AMPure XP beads twice and sequenced by the Illumina platform.

### 2.7. Data Quality Control and Alignment

The adaptor of RNA sequencing data was trimmed by Cutadapt (v1.17, National Bioinformatics Infrastructure Sweden) and the clean reads were aligned to the h19 genome by TopHat (v 2.1.1, Johns Hopkins University, Baltimore, MD, USA). The fragments per kilobase of exon model per million reads mapped (FPKM) was calculated using Cuffnorm (v2.2.1, MATLAB).

The adaptor of PBAT methylation sequencing data was trimmed by TrimGalore (v0.5.0, Babraham Bioinformatics). The first 6 random nucleotides were removed by Cutadapt (v1.17). The clean reads were aligned to mitochondrion DNA using Bismark (v0.22.1, Babraham Bioinformatics). The PCR replicates were removed by SAMtools (v1.9, Genome Research Ltd., Hinxton, UK).

### 2.8. Differentially Expressed Genes (DEGs) Analysis

DEG analysis was performed by the FindAllMarkers command of Seurat (v2.3.4). The DEGs’ expression was visualized by the “ggplot2” package. Gene ontology (GO) enrichment analysis was conducted by DAVID (https://david.ncifcrf.gov/, accessed on 1 October 2016) and the mitochondria-related nuclear genes were selected from MitoCarta3.0 [19]. To simplify visualization of DEGs, we performed with ClusterProfiler gene ontology and Kyoto Encyclopedia of Genes and Genomes (KEGG) analyses to obtain a view of enriched biological pathways that were altered. GO categories were grouped based on “molecular function”, “cellular component”, and “biological processes”.

### 2.9. The CpG Methylation Analysis of Mitochondrion

The average CpG methylation level was calculated and visualized by CGmapTools (v0.1.2, CGmapTools improves the precision of heterozygous SNV calls and supports allele-specific methylation detection and visualization in bisulfite-sequencing data [20], Bioinformatics, 34:381–387.) at a 1000 bp resolution. The global CpG methylation across mitochondrion genes was visualized using Integrative Genomics Viewer (IGV) software (v2.7.2, Broad Institute and the Regents of the University of California, Cambridge, MA, USA).

### 2.10. Statistical Analysis

Statistical analysis was carried out with Prism 8 (GraphPad Software, San Diego, CA, USA). Wilcoxon test was used for gene expression comparison. Correlations were tested by the Spearman’s correlation coefficient. *p* values less than 0.05 were considered statistically significant.

## 3. Results

Two comparisons were performed: the first was between M-IVT and IM-IVT, which was designed to determine the changes in mitochondrial-related nuclear genes during oocyte maturation, and the second was between M-IVT and M-IVO which was aimed to determine the major differences in mitochondrial-related transcriptional profiling.

### 3.1. The Whole Scale of DEGs in Different Comparisons

**Figure 2:**
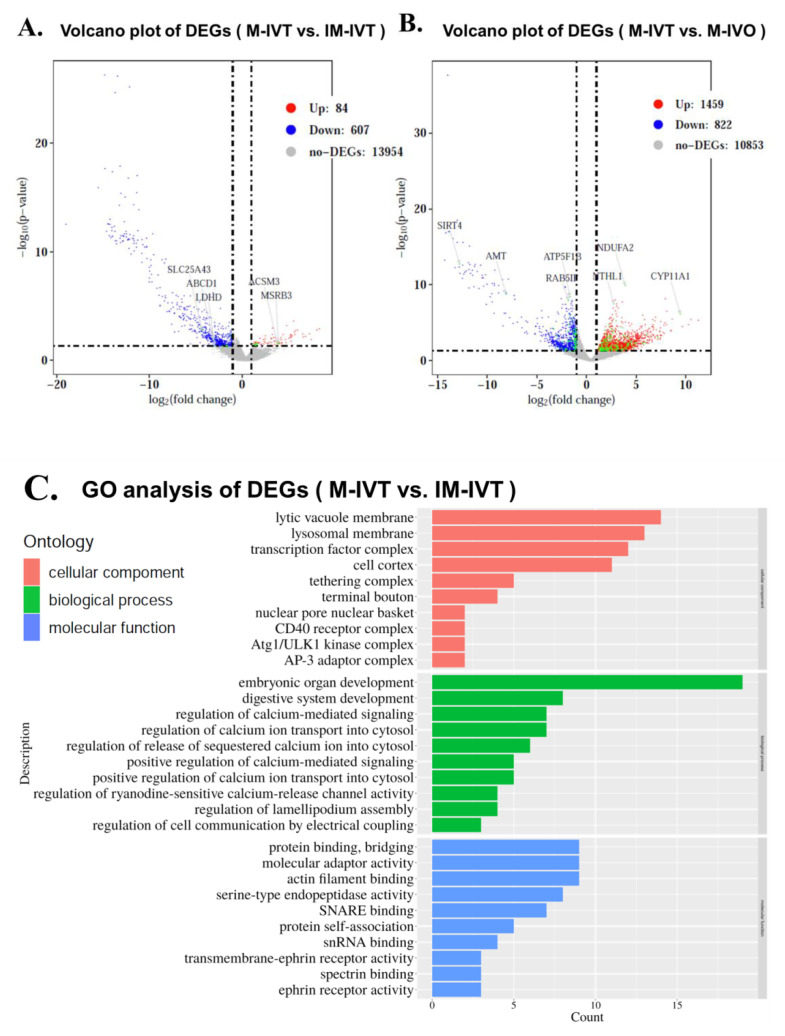

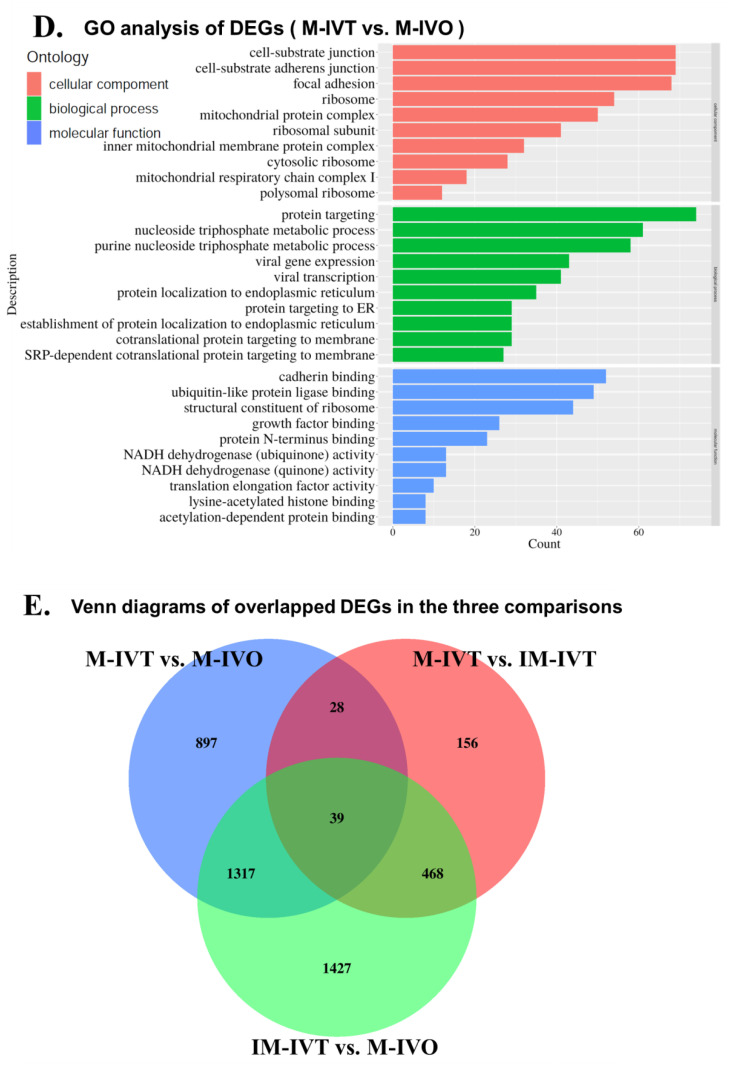
Different scale of DEGs. Volcano plot of differentially regulated genes in oocytes between M-IVT and IM-IVT (**A**), between M-IVT and M-IVO (**B**). Each point in the figures represents a gene. The *X*-axis shows log_2_ (Fold changes) and the *Y*-axis shows −log_10_ (*p*-value). The vertical lines show thresholds for log_2_ ratio larger than 1 or lower than −1. The horizontal line shows threshold for *p*-value <  0.05. The gray points represent gene exhibiting no significant differential expression between IM-IVT and M-IVO or between M-IVT and IM-IVT; the red points represent genes that are upregulated and the blue points represent genes that are downregulated; the green points represent genes that are related to mitochondrion. Gene ontology (GO) categories enrichment analysis of differentially expressed genes in oocytes between M-IVT and IM-IVT (**C**), between M-IVT and M-IVO (**D**). (**E**) Venn diagrams showing the number of DEGs which were overlapped in the three comparisons. 67 (28 + 39) overlapped DEGs were present in the DEGs of M-IVT vs. M-IVO comparison and M-IVT vs. IM-IVT comparison. 1356 (1317 + 39) overlapped DEGs were present in the DEGs of M-IVT vs. M-IVO comparison and M-IVTvs. I M-IVO comparison. 507 (468 + 39) overlapped DEGs were present in the DEGs of M-IVT vs. IM-IVT comparison and IM-IVT vs. M-IVO comparison. 897 DEGs were only present in M-IVT vs. M-IVO comparison. 156 DEGs were only present in M-IVT vs. IM-IVT comparison. 1427 DEGs were only present in IM-IVT vs. M-IVO comparison. 39 overlapped DEGs were present in the DEGs of the three comparisons (M-IVT vs. M-IVO comparison, M-IVT vs. IM-IVT comparison, and IM-IVT vs. M-IVO comparison).

There were 691 DEGs in the first comparison (M-IVT vs. IM-IVT), wherein 84 genes were up-regulated and 607 genes were down-regulated (Figure 2A). A total of 2281 DEGs were identified in the M-IVT oocytes in comparison with M-IVO oocytes; 1459 genes increased while 822 genes decreased (Figure 2B).

To explore the function of DEGs involved in different comparisons, gene ontology (GO) term enrichment analysis was performed. Many significantly enriched dysregulated functions were involved in three categories: cellular component, biological process, and molecular function, respectively. For example, when comparing M-IVT and IM-IVT oocytes, the significant enriched functions linked by the DEGs were cell cortex, lysosomal membrane, lytic vacuole membrane, transcription factor complex, embryonic organ development, actin filament binding, molecular adaptor activity and protein binding, serine-type endopeptidase activity, etc., (Figure 2C). When comparing M-IVT and M-IVO oocytes, the significant enriched functions linked by the DEGs were cell–substrate adherens junction, cell–substrate junction, cytosolic ribosome, focal adhesion, inner mitochondrial membrane protein complex, mitochondrial protein complex, mitochondrial respiratory chain complex I, ribosomal subunit, ribosome, nucleoside triphosphate metabolic process, protein targeting, purine nucleoside triphosphate metabolic process, cadherin binding, NADH dehydrogenase (quinone) activity, NADH dehydrogenase (ubiquinone) activity, structural constituent of ribosome, ubiquitin-like protein ligase binding, etc., (Figure 2D). We then created Venn diagrams in order to visualize the DEG overlaps of the two comparisons. To our surprise, there were 67 overlapped DEGs (Figure 2E and Supplementary Table S1).

### 3.2. The Alterations of Mitochondria-Related Nuclear Genes in the Comparison between M-IVT Group and IM-IVT Group

**Figure 3:**
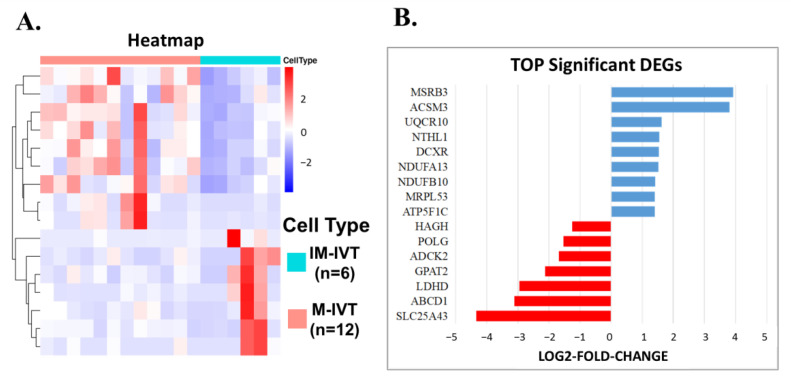

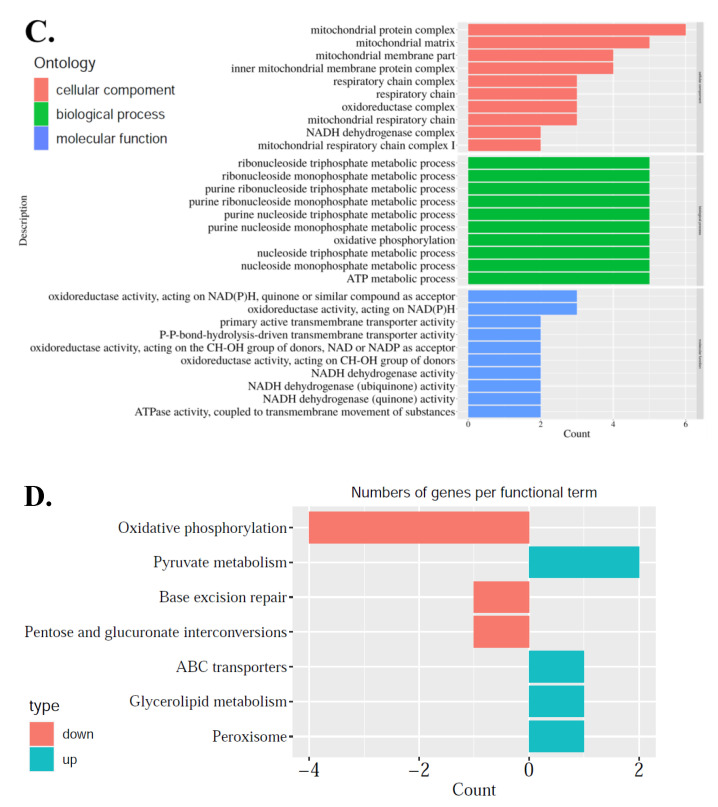
The alterations of mitochondria-related genes in the comparison between M-IVT group and IM-IVT group. (**A**) Hierarchical clustering of differentially expressed genes (DEGs) in oocytes between M-IVT and IM-IVT based on Z-score normalized FPKM values. Each column represents a sample, and each row represents a gene. Blue indicates lower expression and red indicates higher expression. (**B**) Top significant DEGs in oocytes between M-IVT and IM-IVT. (**C**) GO classification of DEGs in oocytes between M-IVT and IM-IVT. *X*-axis represents the GO terms. *Y*-axis represents the number of genes. (**D**) Kyoto Encyclopedia of Genes and Genome analysis of DEGs in oocytes between M-IVT and IM-IVT. *X*-axis represents the counts. *Y*-axis shows the name of the statistical pathway enrichment. Abbreviation: ABCD1, ATP binding cassette subfamily D member 1; ABC transporters: ATP-binding cassette (ABC) transporters; ACSM3, acyl-CoA synthetase medium chain family member 3; ADCK2, aarF domain containing kinase 2; ATP5F1C, ATP synthase F1 subunit gamma; DCXR, dicarbonyl and L-xylulose reductase; GPAT2, glycerol-3-phosphate acyltransferase 2, mitochondrial; HAGH, hydroxyacylglutathione hydrolase; LDHD, lactate dehydrogenase D; MRPL53, mitochondrial ribosomal protein L53; MSRB3, methionine sulfoxide reductase B3; NDUFA13, NADH:ubiquinone oxidoreductase subunit A13; NDUFB10, NADH:ubiquinone oxidoreductase subunit B10; NTHL1, nth like DNA glycosylase 1; POLG, DNA polymerase gamma, catalytic subunit; SLC25A43, solute carrier family 25 member 43; UQCR10, ubiquinol-cytochrome c reductase, complex III subunit X.

For M-IVT vs. IM-IVT oocyte comparisons, 16 mitochondria-related nuclear DEGs were identified, among which 9 were up-regulated and 7 were down-regulated (Figure 3A). Among the top DEGs, MSRB3, and ACSM3 were the most significantly up-regulated genes, and UQCR10, NTHL1, DCXR, NDUFA13, NDUFB10, MRPL53, ATP5F1C were the following up-regulated DEGs. SLC25A43, ABCD1, LDHD, and GPAT2 were the most significantly down-regulated genes and ADCK2, POLG, and HAGH were down-regulated to a lesser extent (Figure 3B).

In addition, GO functional analysis disclosed that the most significantly enriched DEGs were about the structure and biological processes of the mitochondria. For example, the inner mitochondrial membrane, mitochondrial matrix, protein complex, and respiratory chain were involved in the structure of the mitochondria. NADH dehydrogenase complex, oxidoreductase complex, and respiratory chain complex were involved in the biochemical enzyme system. Therefore, the key metabolic processes of ATP, nucleoside, oxidative phosphorylation, purine nucleoside, purine ribonucleoside were altered significantly. However, from the perspective of molecular function, ATPase activity coupled to transmembrane movement of substances, NADH dehydrogenase activity, and oxidoreductase activity were relatively less noticeable (Figure 3C).

Kyoto Encyclopedia of Genes and Genome (KEGG) analysis was performed and revealed that the largest number of differently expressed mitochondria-related genes were involved in the oxidative phosphorylation pathway, while the lowest amount were related to pyruvate metabolism (Figure 3D).

### 3.3. The Alterations of Mitochondria-Related Nuclear Genes in the Comparison between M-IVT Group and M-IVO Group

**Figure 4:**
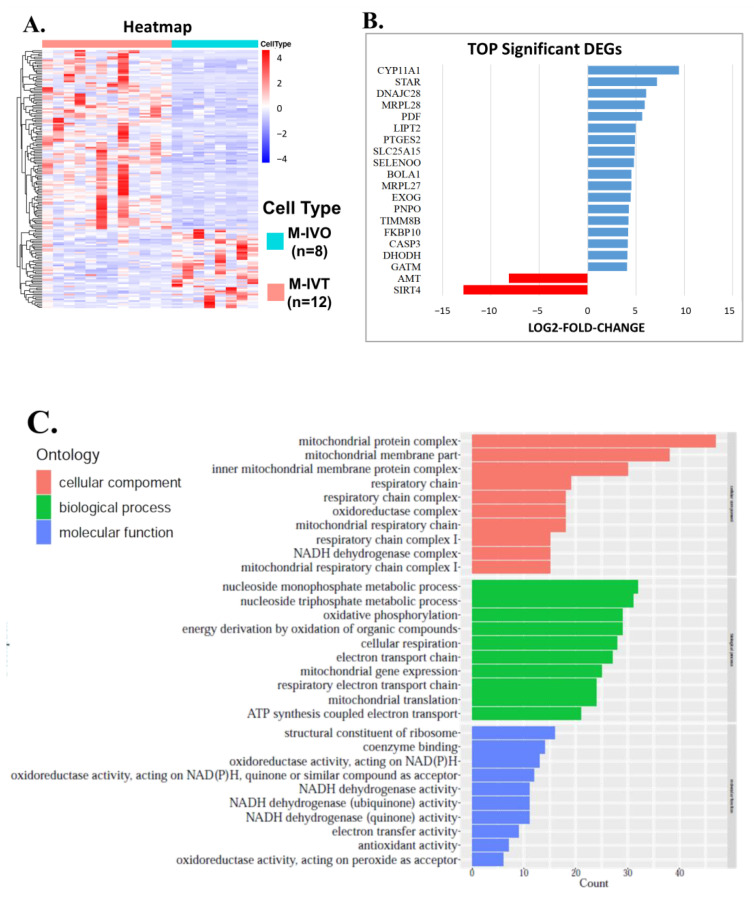

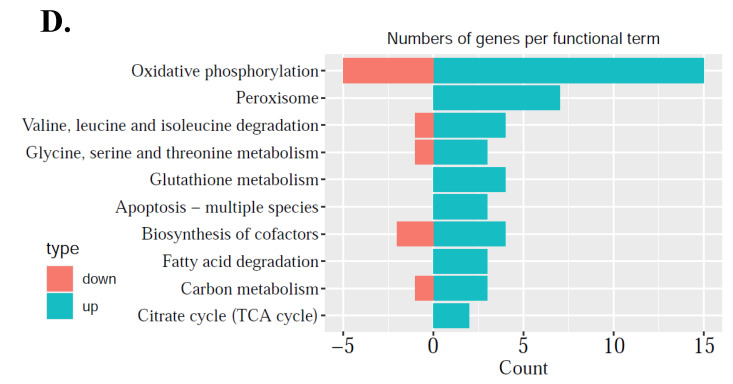
The alterations of mitochondria-related genes in the comparison between M-IVT group and M-IVO group. (**A**) Hierarchical clustering of differentially expressed genes (DEGs) in oocytes between M-IVT and M-IVO based on Z-score normalized FPKM values. Each column represents a sample, and each row represents a gene. Blue indicates lower expression and red indicates higher expression. (**B**) Top significant DEGs in oocytes between M-IVT and M-IVO. (**C**) GO classification of DEGs in oocytes between M-IVT and M-IVO. *X*-axis represents the GO terms. *Y*-axis represents the number of genes. (**D**) Kyoto Encyclopedia of Genes and Genome analysis of DEGs in oocytes between M-IVT and M-IVO. *X*-axis represents the counts. *Y*-axis shows the name of the statistical pathway enrichment. Abbreviation: AMT, aminomethyltransferase; BOLA1, bolA family member 1; CASP3, caspase 3; CYP11A1, cytochrome P450 family 11 subfamily A member 1; DHODH, dihydroorotate dehydrogenase (quinone); DNAJC28, DnaJ heat shock protein family (Hsp40) member C28; EXOG, exo/endonuclease G; FKBP10, FKBP prolyl isomerase 10; GATM, glycine amidinotransferase; LIPT2, lipoyl(octanoyl) transferase 2; MRPL27, mitochondrial ribosomal protein L27; MRPL28, mitochondrial ribosomal protein L28; PDF, peptide deformylase, mitochondrial; PNPO, pyridoxamine 5’-phosphate oxidase; PTGES2, prostaglandin E synthase 2; SELENOO, selenoprotein O; SIRT4, sirtuin 4; SLC25A15, solute carrier family 25 member 15; STAR, steroidogenic acute regulatory protein; TIMM8B, translocase of inner mitochondrial membrane 8 homolog B.

As the key roles of mitochondria for oocyte maturation, not only in vitro, but also in vivo, we focused on changes in mitochondria-related genes, among which 111 were up-regulated and 49 were down-regulated when comparing the M-IVT group to the M-IVO group (Figure 4A). The top differently expressed mitochondria-related nuclear genes are shown in Figure 4B, with the most significantly increased genes being CYP11A1, STAR, DNAJC28, MRPL28, PDF, and so on, and the most significantly decreased genes being SIRT4 and AMT.

GO function enriched for these mitochondria-related nuclear DEGs was performed. The major biological events were about the cellular component and biological processes (Figure 4C). For the mitochondrial structure, relevant DEGs were related to inner mitochondrial membrane protein complex, mitochondrial matrix, mitochondrial protein complex, and respiratory chain complex I. Corresponding to these DEGs, the dysregulation was mainly about cellular respiration, electron transport chain, oxidative phosphorylation, energy derivation by oxidation of organic compounds, mitochondrial gene express, and mitochondrial translation.

KEGG analysis of mitochondria-related genes showed that the most significant differences were involved in pathways such as oxidative phosphorylation, thermogenesis, aminoacyl-tRNA biosynthesis. Nicotinate and nicotinamide metabolism also took part in the dysregulation (Figure 4D).

### 3.4. The Common Altered Mitochondria-Related Nuclear Genes in Three Different Kinds of Oocytes

**Figure 5:**
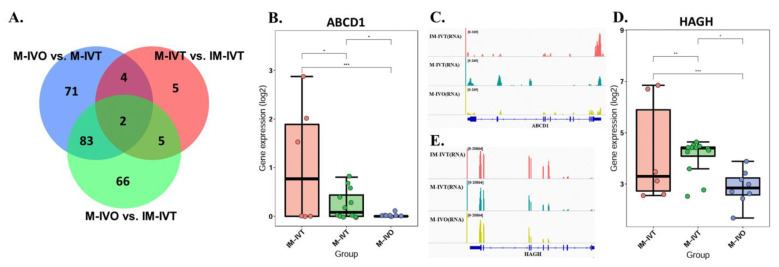
The common altered mitochondria-related genes in the comparisons. (**A**) Venn diagrams showing the number of mitochondria-related genes, which were overlapped within the three comparisons. 2 overlapped mitochondria-related DEGs were present in the DEGs of the three comparisons (M-IVO vs. M-IVT comparison, M-IVT vs. IM-IVT comparison, and M-IVO vs. IM-IVT comparison). 6 overlapped mitochondria-related DEGs were present in the DEGs of M-IVO vs. M-IVT comparison and M-IVT vs. IM-IVT comparison. 85 overlapped mitochondria-related DEGs were present in the DEGs of M-IVT vs. M-IVO comparison and M-IVO vs. IM-IVT comparison. 7 overlapped mitochondria-related DEGs were present in the DEGs of M-IVT vs. IM-IVT comparison and M-IVO vs. IM-IVT comparison. 71 mitochondria-related DEGs were only present in M-IVO vs. M-IVT comparison. 5 mitochondria-related DEGs were only present in M-IVT vs. IM-IVT comparison. 66 mitochondria-related DEGs were only present in M-IVO vs. IM-IVT comparison. The gene transcript expression level in oocytes from IM-IVT group, M-IVT group and M-IVO group for ABCD1 (**B**) and HAGH (**D**). Browser view of RNA-seq signals on the gene ABCD1 (**C**) and HAGH (**E**) in IM-IVT, M-IVT and M-IVO group. The scale of normalized reads is shown for the RNA-seq data. *** *p* < 0.001, ** *p* < 0.01 and * *p* < 0.05 by Wilcoxon test (B and D). Abbreviation: ABCD1, ATP binding cassette subfamily D member 1; HAGH, hydroxyacylglutathione hydrolase.

Venn diagrams were created in order to determine the same genes that were altered in these three different states (Figure 5A). ABCD1 and HADH were the only two genes which were present in the DEGs of the three comparisons. A significant increase in ABCD1 mRNA expression level was observed in oocytes from the M-IVT group compared to those from M-IVO (Figure 5B). Furthermore, the mRNA expression level of ABCD1 was significantly higher in oocytes from IM-IVT than in oocytes from either M-IVT or M-IVO (Figure 5B). This was also the case for HAGH. The mRNA expression level was significantly increased in oocytes from the M-IVT group compared to oocytes from the M-IVO group (Figure 5D). Additionally, the HAGH mRNA expression level was enhanced in oocytes from IM-IVT compared to oocytes from M-IVT or M-IVO (Figure 5D).

### 3.5. Increased mRNA Expression Level of PHB, GPX4, and PNPO Were Correlated with the Methylation Level of their Promoter Region

**Figure 6:**
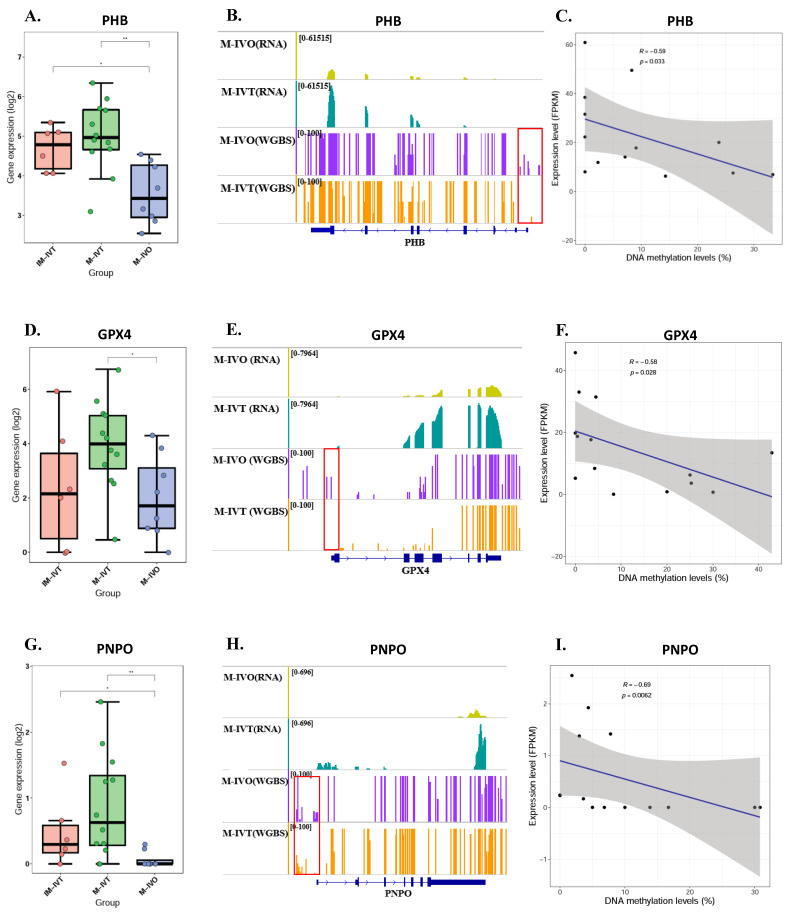
Increased mRNA expression level of PHB, GPX4, and PNPO were correlated with the methylation level of their promoter region. The gene transcript expression level of PHB (**A**), GPX4 (**D**), and PNPO (**G**) in oocytes from IM-IVT group, M-IVT group and M-IVO group. Browser view of RNA-seq and bisulfite-seq signals on the gene PHB (**B**), GPX4 (**E**), and PNPO (**H**) in the IM-IVT and M-IVO. The scale of normalized reads is shown for the RNA-seq data. The scale of DNA methylation ratio is shown for the bisulfite-seq data. For PHB (**C**), GPX4 (**F**), and PNPO (**I**), DNA methylation levels is plotted on the *X*-axis, while gene expression level (FPKM) is plotted on the *Y*-axis. ** *p* < 0.01 and * *p* < 0.05 by Wilcoxon test (**A**,**D**,**G**). Abbreviation: GPX4, glutathione peroxidase 4; PHB, prohibitin; PNPO, pyridoxamine 5’-phosphate oxidase.

There was a significant increase in the mRNA expression level of PHB, GPX4, and PNPO in M-IVT group compared to M-IVO group (Figure 6A,D,G). Additionally, RNA-seq signals and bisulfite-seq signals indicating methylation level on the gene in the M-IVT and M-IVO from our previous report [13] can be clearly visualized by the IGV program [21] (Figure 6B,E,H). There was a significant negative relationship between gene promoter methylation level and mRNA expression level of the gene in oocytes from M-IVT and M-IVO (PHB: R = −0.59, *p* = 0.003, Figure 6C; GPX4: R = −0.58, *p* = 0.028; Figure 6F; PNPO: R = −0.69, *p* = 0.0062; Figure 6I), suggesting that the decreased methylation level might be a cause of increased mRNA expression level in oocytes from M-IVT compared to those from the M-IVO group.

## 4. Discussion

Human oocyte growth and maturation has three states: GV stage, MI stage, and MII stage from the morphology and dynamics of chromosomes. This process encompasses the completion of the 1st asymmetric meiotic division and accompanying process essential for subsequent oocyte activation, formation of pronuclei, and preimplantation embryogenesis. In brief, the process consists of two major events: nuclear maturation and the cytoplasmic maturation [22]. Both the symbolic events of germinal vesicle breakdown (GVBD) and extrusion of the 1st polar body are regarded as nuclear maturation. Normally, the two aspects are a coordinated event, with GVBD releasing nuclear contents into oocyte cytoplasm. This undoubtedly has some effects on cytoplasmic maturation, at least in part. However, events comprising cytoplasmic maturation are less well defined than those comprising nuclear maturation. This is the way to solve the suboptimal developmental potential of in vitro matured oocytes in contrast to in vivo matured oocytes. In addition, it is rational to understand how mitochondria-related nuclear genes are altered during oocyte maturation. Several studies focused on the mitochondria’s role in cytoplasmic maturation and attempted to disclose its mechanisms, owing to (1) ATP generated by the mitochondria is necessary for both nuclear meiotic maturation and subsequent cytokinesis; (2) explicit changes in mitochondrial distribution during human oocyte maturation have been demonstrated by several studies [14,23]. However, the morphological dynamic distribution of mitochondria in human oocytes during the process of maturation should be further explored with respect to functions. Coticchio et al. observed the ultrastructure of human IVM oocytes using transmission electron microscopy, and they found that the mitochondrial morphology in IVM oocytes was similar with the mitochondrial morphology in in vivo matured oocytes, but large mitochondria–vesicle complexes partially replaced mitochondria–smooth endoplasmic reticulum aggregates in IVM oocytes [12]. This supported the fact that mitochondria participated in the process of cytoplasmic maturation. Our present work compared three oocyte states: in vivo matured oocytes, in vitro matured oocytes after IVM operation, and immature oocytes. Through comparison, this study aimed at (1) disclosure of the changes in mitochondria-related nuclear genes and metabolic pathway in the process of oocyte maturation; (2) analysis of the detailed differences on mitochondria regulation between the two kinds of matured oocytes.

First, from a comparison between matured and immature oocytes in vitro, 691 DEGs were identified. After careful analysis, the difference was mainly the focus on abnormal cytoplasmic maturation, such as cell cortex, lysosomal membrane, lytic vacuole membrane, transcription factor complex, embryonic organ development, actin filament binding, molecular adaptor activity and protein binding, serine-type endopeptidase activity, etc. Unfortunately, until now, there has been no clear definition and precise clinical assessment criteria for cytoplasmic maturity [24,25]. As maturation is a prerequisite for oocytes to accumulate enough energy and nutrition to support post-fertilization stage of development, this study addresses the importance of mitochondrial regulation alterations during oocyte maturation, so mitochondria-related DEGs were further explored. These DEGs were related to mitochondrial structure and function: genes associated with mitochondrial inner membrane structure, mitochondrial respiratory chain complex I, and NADH dehydrogenase complex were up-regulated, which meant that the structure of the mitochondria needs to be adjusted in the process of oocyte maturation. As the most important functions of energy metabolism, oxidative phosphorylation, base excision repair, pentose and glucuronate interconversion were down-regulated, while pyruvate metabolism, ABC transporters, glycerolipid metabolism, and peroxisome were up-regulated (Figure 3D). This phenomenon showed that the main source of ATP shifted from glucose metabolism to pyruvate and fatty acid oxidation during oocyte maturation in order to maintain a low level of otherwise damaging reactive oxygen species (ROS) production. The latter can cause oxidative damage, targeting proteins, such as DNA, including both nuclear DNA and mitochondrial DNA (mtDNA) due to their highly oxidative nature. Meanwhile two DEGs were identified in three comparisons: the first was ABCD1, ATP binding cassette subfamily D member 1. The protein (ABC) encoded by this gene was a member of the superfamily of ATP-binding cassette transporters. ABC proteins transported various molecules across extra- and intra-cellular membranes and were involved in peroxisomal import or catabolism of fatty acids and/or fatty acyl-CoAs in organelles. Evidence in mice documented that this ABC protein was expressed on the mitochondrional membrane in oocytes and illustrated that dysfunctional ABCD1 disrupts mitochondrial homeostasis [26]. The other was HAGH, which encoded the enzyme responsible for the hydrolysis of S-lactoyl-glutathione to reduce glutathione and D-lactate. Both represent the key regulators for cytoplasmic maturation, which have been recognized as fundamental for oocyte maturation.

Second, since nuclear encoded mitochondrial proteins may cause mitochondrial dysfunction of energy production and mitochondrial disorders, it is necessary to compare in vitro matured oocytes to in vivo matured oocytes to improve IVM techniques. From the comparison between M-IVT oocytes and M-IVO oocytes, 2281 DEGs were identified. Many significant enriched functions were involved in cellular component, biological process, and molecular function categories. For instance, in contrast to M-IVO, M-IVT oocytes were very different with respect to cell–substrate adherens junction, cell–substrate junction, focal adhesion, and protein targeting. These biological dysfunctions are related to the survival and morphogenesis of an embryonic lineage upon implantation and pluripotency transition [27,28], which may be the reason why the implantation rate of embryos derived from IVM oocyte is lower than that of embryos from in vivo matured oocytes in clinical applications. Regarding mitochondria-related gene expression, there were 160 DEGs that were mitochondria related, including 111 that were up-regulated and 49 that were down-regulated. These DEGs participated in oxidative phosphorylation, peroxisome, valine leucine and isoleucine degradation, glycine serine and theronine metabolism, glutathione metabolism, apoptosis, biosynthesis of cofactors, fatty acid degradation, carbon metabolism, and the citrate cycle (TCA cycle). The most significant difference was oxidative phosphorylation, which implied that the function of mitochondria was regulated by the nuclear gene to compensate for the developmental need in the process of oocyte maturation using IVM techniques. Thus, this study indicates that the IVM culture medium should be optimized according to this information, as it is a latent strategy to improve the effectiveness of IVM procedures though regulating mitochondrial biological reactions. Recently, it was reported that supplementation of melatonin in IVM culture medium can promote and improve the development of human oocytes matured in vitro, and this study discovered that the mechanisms of the beneficial effect were mediated by increasing the mitochondrial membrane potential and decreasing intracellular ROS and Ca^2+^ levels [29]. In addition, Min et al. used *Caenorhabditis elegans* as a model to demonstrate that supplementation of nicotinamide can reverse the decline of oocyte quality and improve offspring development in aged oocytes through restoring mitochondrial membrane potential and reducing the level of reactive oxygen species [30]. Other evidence in mouse showed that modulation of mitochondrial redox balance by supplementation of nicotinamide mononucleotide can reverse the declining quality of maternally aged oocytes (published in 2020) [31]. However, so far as we know, there is no report that addresses modification of IVM culture medium through supplementation of nicotinamide or nicotinamide mononucleotide to promote the potential of matured human oocytes in vitro.

The third and equally crucial question was about epigenetics, post translational modification of genes associated with mitochondrial function. Our results did not show that methylation levels of mitochondrial-associated genes had significantly changed between M-IVT and M-IVO oocytes. However, the methylation levels of three genes, PHB1, GPX4, and PNPO, were significantly reduced and negatively correlated with corresponding RNA levels. These correlations could be the cause of the changes in transcription levels. For example, the reason why the RNA expression of GPX4, glutathione peroxidase 4 did increase in M-IVT oocytes compared to M-IVO oocytes was that the protein encoded by this gene catalyzed the reduction of hydrogen peroxide, organic hydroperoxides, and lipid hydroperoxides, and thereby protected cells against oxidative damage. This isozyme has a high preference for lipid hydroperoxides and protects cells against membrane lipid peroxidation and cell death. Thus, this phenomenon might be an adaptive adjustment of oocytes themselves to oxidative stress changes in the process of in vitro maturation, implying that the IVM medium should be modified according to actual requirements.

There are also some limitations to this study: 1). Due to the rarity of human resources, the sample size was relatively small, which could limit further validation using the other techniques, such as RT-qPCR. 2). Furthermore, we did not detect the methylation levels of genes in IM-IVT group, because the transcriptomic dynamics might be regulated by genomic DNA methylation, histone acetylation, microRNA-induced RNA degradation, etc., [32,33,34,35]. Therefore, it would be a valuable future direction to explore the possible mechanism which regulates the DEGs in the biological process of oocyte maturation.

## 5. Conclusions

In conclusion, our study revealed some significant DEG changes in the process of oocyte maturation via single-cell analysis and found that these changes are involved in cellular structure, cell cycle progression, nutrient metabolism, and energy production. We identified 16 mitochondria-related nuclear DEGs and disclosed the correlated alteration in the metabolic pathway, which showed that the main source of ATP shifted from glucose metabolism to pyruvate and fatty acid oxidation during oocyte maturation in order to maintain a low level of damaging reactive oxygen species (ROS) production. Meanwhile, by comparing in vitro matured oocytes with in vivo matured oocytes, the most significant difference was oxidative phosphorylation, which implied that the function of mitochondria was regulated by nuclear gene to compensate for the developmental need in the process of oocyte maturation from IVM techniques. Thus, this study indicated that the IVM culture medium should be optimized according to this information, as it is a latent strategy to improve the effectiveness of IVM procedures though regulating mitochondrial biological reactions.

## Data Availability

Further information and requests for data should be directed to and will be fulfilled by the Lead Contact, Ri-Cheng Chian (rchian@126.com).

## Supplementary Materials

The following supporting information can be downloaded at: https://www.mdpi.com/article/10.3390/cells11020297/s1, Table S1: List of overlapped DEGs in comparison (M-IVT vs. M-IVO) and comparison (M-IVT vs. IM-IVT).
