# Supplementary material for: Changes in the Mitochondria-Related Nuclear Gene Expression Profile during Human Oocyte Maturation by the IVM Technique
Source: Cells. 2022 Jan 16;11(2):297. doi: 10.3390/cells11020297 (PMC8774259; doi:10.3390/cells11020297)
Supplement: Supplementary file 1 [file cells-11-00297-s001.zip › cells-1506836-supplementary.pdf]

Table S1: List of overlapped DEGs in comparison (M-IVT *vs* M-IVO) and comparison (M-IVT *vs* IM-IVT).

Gene ID	Gene Name	Annotation	Class
ENSG00000101986	ABCD1	ATP binding cassette subfamily D member 1 [Source:HGNC Symbol;Acc:HGNC:61]	protein_coding
ENSG00000136379	ABHD17C	abhydrolase domain containing 17C [Source:HGNC Symbol;Acc:HGNC:26925]	protein_coding
ENSG00000237729	AC002075.2	ribosomal protein S27 (RPS27) pseudogene	processed_pseudogene
ENSG00000244563	AC006011.1	ribosomal protein S26 (RPS26) pseudogene	processed_pseudogene
ENSG00000266977	AC006504.2	novel transcript	lincRNA
ENSG00000283549	AC007161.3	novel transcript	processed_transcript
ENSG00000259855	AC007749.1	novel transcript	lincRNA
ENSG00000267607	AC011511.5	novel transcript, antisense to ICAM4 and ICAM1	antisense
ENSG00000267053	AC012617.1	novel transcript	lincRNA
ENSG00000275097	AC024940.6	novel transcript, overlapping 3'UTR of FAM60A	3prime_overlapping_ncRNA
ENSG00000272323	AC026801.2	novel transcript, antisense to TTC23L	antisense
ENSG00000261030	AC079171.1	novel transcript	lincRNA
ENSG00000256923	AC084819.1	novel transcript	lincRNA
ENSG00000245059	AC092718.1	novel transcript	lincRNA
ENSG00000110711	AIP	aryl hydrocarbon receptor interacting protein [Source:HGNC Symbol;Acc:HGNC:358]	protein_coding
ENSG00000131503	ANKHD1	ankyrin repeat and KH domain containing 1 [Source:HGNC Symbol;Acc:HGNC:24714]	protein_coding
ENSG00000198720	ANKRD13B	ankyrin repeat domain 13B [Source:HGNC Symbol;Acc:HGNC:26363]	protein_coding

ENSG00000134461	ANKRD16	ankyrin repeat domain 16 [Source:HGNC Symbol;Acc:HGNC:23471]	protein_coding
ENSG00000267249	AP005482.3	novel transcript, sense intronic CEP76	sense_intronic
ENSG00000169696	ASPSCR1	ASPSCR1, UBX domain containing tether for SLC2A4 [Source:HGNC Symbol;Acc:HGNC:13825]	protein_coding
ENSG00000235106	BRD3OS	BRD3 opposite strand [Source:HGNC Symbol;Acc:HGNC:24742]	protein_coding
ENSG00000169738	DCXR	dicarbonyl and L-xylulose reductase [Source:HGNC Symbol;Acc:HGNC:18985]	protein_coding
ENSG00000158856	DMTN	dematin actin binding protein [Source:HGNC Symbol;Acc:HGNC:3382]	protein_coding
ENSG00000163840	DTX3L	deltex E3 ubiquitin ligase 3L [Source:HGNC Symbol;Acc:HGNC:30323]	protein_coding
ENSG00000063245	EPN1	epsin 1 [Source:HGNC Symbol;Acc:HGNC:21604]	protein_coding
ENSG00000104884	ERCC2	ERCC excision repair 2, TFIIH core complex helicase subunit [Source:HGNC Symbol;Acc:HGNC:3434]	protein_coding
ENSG00000242950	ERVW-1	endogenous retrovirus group W member 1, envelope [Source:HGNC Symbol;Acc:HGNC:13525]	protein_coding
ENSG00000283632	EXOC3L2	exocyst complex component 3 like 2 [Source:HGNC Symbol;Acc:HGNC:30162]	protein_coding
ENSG00000135842	FAM129A	family with sequence similarity 129 member A [Source:HGNC Symbol;Acc:HGNC:16784]	protein_coding
ENSG00000063854	HAGH	hydroxyacylglutathione hydrolase [Source:HGNC Symbol;Acc:HGNC:4805]	protein_coding
ENSG00000125968	ID1	inhibitor of DNA binding 1, HLH protein [Source:HGNC Symbol;Acc:HGNC:5360]	protein_coding

ENSG00000171097	KYAT1	kynurenine aminotransferase 1 [Source:HGNC Symbol;Acc:HGNC:1564]	protein_coding
ENSG00000240567	LINC02067	long intergenic non-protein coding RNA 2067 [Source:HGNC Symbol;Acc:HGNC:52913]	lincRNA
ENSG00000103227	LMF1	lipase maturation factor 1 [Source:HGNC Symbol;Acc:HGNC:14154]	protein_coding
ENSG00000164877	MICALL2	MICAL like 2 [Source:HGNC Symbol;Acc:HGNC:29672]	protein_coding
ENSG00000267532	MIR497HG	mir-497-195 cluster host gene [Source:HGNC Symbol;Acc:HGNC:39523]	antisense
ENSG00000174099	MSRB3	methionine sulfoxide reductase B3 [Source:HGNC Symbol;Acc:HGNC:27375]	protein_coding
ENSG00000140990	NDUFB10	NADH:ubiquinone oxidoreductase subunit B10 [Source:HGNC Symbol;Acc:HGNC:7696]	protein_coding
ENSG00000130305	NSUN5	NOP2/Sun RNA methyltransferase family member 5 [Source:HGNC Symbol;Acc:HGNC:16385]	protein_coding
ENSG00000065057	NTHL1	nth like DNA glycosylase 1 [Source:HGNC Symbol;Acc:HGNC:8028]	protein_coding
ENSG00000184792	OSBP2	oxysterol binding protein 2 [Source:HGNC Symbol;Acc:HGNC:8504]	protein_coding
ENSG00000069943	PIGB	phosphatidylinositol glycan anchor biosynthesis class B [Source:HGNC Symbol;Acc:HGNC:8959]	protein_coding
ENSG00000164087	POC1A	POC1 centriolar protein A [Source:HGNC Symbol;Acc:HGNC:24488]	protein_coding
ENSG00000147231	RADX	RPA1 related single stranded DNA binding protein, X-linked [Source:HGNC Symbol;Acc:HGNC:25486]	protein_coding
ENSG00000115963	RND3	Rho family GTPase 3 [Source:HGNC	protein_coding

		Symbol;Acc:HGNC:671]	
ENSG00000092098	RNF31	ring finger protein 31 [Source:HGNC Symbol;Acc:HGNC:16031]	protein_coding
ENSG00000182383	RPL27AP5	ribosomal protein L27a pseudogene 5 [Source:HGNC Symbol;Acc:HGNC:36420]	processed_pseudogene
ENSG00000232134	RPS15AP12	ribosomal protein S15a pseudogene 12 [Source:HGNC Symbol;Acc:HGNC:36759]	processed_pseudogene
ENSG00000213363	RPS3P6	ribosomal protein S3 pseudogene 6 [Source:HGNC Symbol;Acc:HGNC:36061]	processed_pseudogene
ENSG00000172426	RSPH9	radial spoke head 9 homolog [Source:HGNC Symbol;Acc:HGNC:21057]	protein_coding
ENSG00000130766	SESN2	sestrin 2 [Source:HGNC Symbol;Acc:HGNC:20746]	protein_coding
ENSG00000198964	SGMS1	sphingomyelin synthase 1 [Source:HGNC Symbol;Acc:HGNC:29799]	protein_coding
ENSG00000104969	SGTA	small glutamine rich tetratricopeptide repeat containing alpha [Source:HGNC Symbol;Acc:HGNC:10819]	protein_coding
ENSG00000197847	SLC22A20P	solute carrier family 22 member 20, pseudogene [Source:HGNC Symbol;Acc:HGNC:29867]	transcribed_unitary_pseudogene
ENSG00000109062	SLC9A3R1	SLC9A3 regulator 1 [Source:HGNC Symbol;Acc:HGNC:11075]	protein_coding
ENSG00000011347	SYT7	synaptotagmin 7 [Source:HGNC Symbol;Acc:HGNC:11514]	protein_coding
ENSG00000166575	TMEM135	transmembrane protein 135 [Source:HGNC Symbol;Acc:HGNC:26167]	protein_coding
ENSG00000007255	TRAPPC6A	trafficking protein particle complex 6A [Source:HGNC Symbol;Acc:HGNC:23069]	protein_coding

ENSG00000100304	TTLL12	tubulin tyrosine ligase like 12 [Source:HGNC Symbol;Acc:HGNC:28974]	protein_coding
ENSG00000104142	VPS18	VPS18, CORVET/HOPS core subunit [Source:HGNC Symbol;Acc:HGNC:15972]	protein_coding
ENSG00000149823	VPS51	VPS51, GARP complex subunit [Source:HGNC Symbol;Acc:HGNC:1172]	protein_coding
ENSG00000163159	VPS72	vacuolar protein sorting 72 homolog [Source:HGNC Symbol;Acc:HGNC:11644]	protein_coding
ENSG00000174776	WDR49	WD repeat domain 49 [Source:HGNC Symbol;Acc:HGNC:26587]	protein_coding
ENSG00000285531	Z83840.1	LLP homolog, long-term synaptic facilitation (LLPH) pseudogene	processed_pseudogene
ENSG00000204859	ZBTB48	zinc finger and BTB domain containing 48 [Source:HGNC Symbol;Acc:HGNC:4930]	protein_coding
ENSG00000179922	ZNF784	zinc finger protein 784 [Source:HGNC Symbol;Acc:HGNC:33111]	protein_coding
ENSG00000174276	ZNHIT2	zinc finger HIT-type containing 2 [Source:HGNC Symbol;Acc:HGNC:1177]	protein_coding
